# Columnaris disease in fish: a review with emphasis on bacterium-host interactions

**DOI:** 10.1186/1297-9716-44-27

**Published:** 2013-04-24

**Authors:** Annelies Maria Declercq, Freddy Haesebrouck, Wim Van den Broeck, Peter Bossier, Annemie Decostere

**Affiliations:** 1 Department of Morphology, Faculty of Veterinary Medicine, Ghent University, Merelbeke, Belgium; 2 Department of Pathology, Bacteriology and Avian Diseases, Faculty of Veterinary Medicine, Ghent University, Merelbeke, Belgium; 3 Laboratory of Aquaculture and Artemia Reference Center, Ghent University, Ghent, Belgium

## Abstract

*Flavobacterium columnare* (*F. columnare*) is the causative agent of columnaris disease. This bacterium affects both cultured and wild freshwater fish including many susceptible commercially important fish species. *F. columnare* infections may result in skin lesions, fin erosion and gill necrosis, with a high degree of mortality, leading to severe economic losses. Especially in the last decade, various research groups have performed studies aimed at elucidating the pathogenesis of columnaris disease, leading to significant progress in defining the complex interactions between the organism and its host. Despite these efforts, the pathogenesis of columnaris disease hitherto largely remains unclear, compromising the further development of efficient curative and preventive measures to combat this disease. Besides elaborating on the agent and the disease it causes, this review aims to summarize these pathogenesis data emphasizing the areas meriting further investigation.

## Table of contents

1. The agent

1.1 History and taxonomy

1.1 Morphology and biochemical characteristics

1.1 Epidemiology

2. The disease

2.1 Clinical signs, histopathology, ultrastructural features and haematology

2.1 Diagnosis

3. Pathogenesis

3.1 Colonization

3.1 Exotoxins, bacteriocins and endotoxins

3.1 Interaction with the fish immune system

4. Importance of environmental factors

5. Experimental trials/pathogenicity tests

6. Control

6.1 Preventive measures

6.1 Curative approach

7. Conclusion

8. Competing interests

9. Authors’ contributions

10. Acknowledgements

11. References

## 1. The agent

### 1.1 History and taxonomy

*Flavobacterium columnare* (*F. columnare*), the causative agent of columnaris disease, belongs to the family *Flavobacteriaceae*[1-3]. Columnaris disease was first described by Davis among warm water fishes from the Mississippi River [4]. Although unsuccessful in cultivating the etiological agent, Davis described the disease and reported large numbers of slender, motile bacteria present in the lesions [4]. Upon examining a wet mount preparation of these lesions, column-like structures formed by these bacteria were evident. The organism was hence named *Bacillus columnaris* and the disease elicited columnaris disease. Over the decades the taxonomic status of the pathogen has changed several times since the pioneering work of Davis [4]. In 1944, Ordal and Rucker were the first to isolate the bacterium from a natural outbreak of columnaris disease among sockeye salmon (*Onchorhynchus nerka*) [5]. A diluted culture medium was used to grow the bacterium. Based on cellular morphology, they identified the bacterium as a myxobacterium. Organisms classified in the order *Myxobacteria* are long, thin Gram-negative rods that are motile on agar media by a creeping or flexing motion. They have a life cycle composed of vegetative cells, microcysts (resting cells), and fruiting bodies, or only vegetative cells and microcysts [6]. Ordal and Rucker reported that the myxobacterium from columnaris disease produced both fruiting bodies and microcysts and named the organism *Chondrococcus columnaris*[5]. Garnjobst assigned the bacterium to the family *Cytophagaceae* as *Cytophaga columnaris* after isolating a pathogenic bacterium resembling *Chondrococcus columnaris* morphologically, but not producing microcysts [7]. Bernardet and Grimont reclassified the organism and placed it in the family *Cytophagaceae* and the genus *Flexibacter*, assigning it as *Flexibacter columnaris*[8]. Finally, in 1996, the bacterium received its current name, *Flavobacterium columnare*, based on DNA-rRNA hybridization data and protein and fatty acid profiles [3]. In 1999, the *F. columnare* cluster was subdivided in three genomovars based on differences in 16S rRNA sequences, restriction fragment length polymorphism (RFLP) and DNA-DNA-hybridization [9].

### 1.2 Morphology and biochemical characteristics

The morphological and biochemical characteristics of *F. columnare* are summarized in Table 1. For a full biochemical profile of *F. columnare*, the reader is referred to Bernardet and Bowman [1].

**Table 1:** **Morphological and biochemical characteristics of *F. columnare *(adapted from**[1,3,10-12]**)**

**Characteristic**	**Description**
Growth condition	Strictly aerobic
Gram-stain	Gram-negative
Morphology	Long, slender gliding rods of 4 to 10 μm and 0.3 to 0.5 μm wide. In aging cultures spheroplasts may occur
Capsule	Described to be absent [10] or present [11] depending on the adopted strain
Congo red absorption	Present due to an extracellular galactosamine glycan in the mucus and the production of flexirubin-type pigments
H_2_S-production	Present
Degradation of crystalline cellulose	Absent
Degradation of complex acidic polysaccharides of connective tissue	Present

### 1.3 Epidemiology

*F. columnare* is distributed worldwide in fresh water sources and may infect many different wild and cultured freshwater fish species, such as (but not limited to) carp, channel catfish, goldfish, eel, perch, salmonids and tilapia [1,12-17]. This disease also assails many tropical freshwater aquarium fish [12,14]. In the channel catfish (*Ictalurus punctatus*) industry in the United States, *F. columnare* is the second most prevalent bacterium, after *Edwardsiella ictaluri*, to cause disease and mortality [18,19], with yearly losses estimated at 30 million dollars [20]. This organism can also be part of the bacterial microbiota of freshwater fish, eggs and the rearing waters the fish live in [21]. Fish may reside in a clinically healthy carrier status harbouring an isolate remaining from a previous outbreak of columnaris disease and in this way act as an infection source for other fish [22,23]. Fujihara and Nakatani reported that rainbow trout surviving a *F. columnare* infection can release up to 5 × 10^3^ colony forming units/mL/h of viable bacteria into tankwater [22]. The gills were shown to be the major release site of this pathogen. Dead fish would be able to spread the disease at a higher transmission rate compared to living fish [24].

Several studies have indicated the potential for *F. columnare* to survive for extended periods in water. Survival was demonstrated to be influenced by physical and chemical characteristics of the surrounding water. Fijan indicated that *F. columnare* can survive up to 16 days at 25°C in hard, alkaline water with a high organic load [25]. Soft water with 10 mg/L CaCO_3_, especially when acid or with a low organic content, does not provide a favorable environment for the organism [25]. Chowdhury and Wakabayashi determined that calcium, magnesium, potassium and sodium ions all are important for long-term survival of *F. columnare* in water [26]. Ross and Smith found that survival of *F. columnare* in static, sterile river water was directly related to temperature, with a higher survival percentage at lower temperatures [27]. The bacterium can keep its infectivity in lake water in laboratory conditions for at least five months [28]. *F. columnare* is also capable of surviving in sterile river mud [6]. Apparently, mud slurry often contains sufficient nutrients to maintain viability of *F. columnare* longer than sterile river water. In this case, however, the percentage survival of *F. columnare* seeded into mud seems to be higher at 25°C than at 5°C. Temperatures below 5°C are even detrimental to *F. columnare* cells in mud. *F. columnare* also grows well on particulate fish feed [29]. When surviving outside the host, *F. columnare* can change from a virulent to a less virulent form with an altered colony morphology, probably to save energy [28]. It has been suggested that *F. columnare* strains at fish farms originate from environmental waters and that the farm environment and practices may select for virulent strains that cause outbreaks in the farm [28,30].

## 2. The disease

### 2.1 Clinical signs, histopathology, ultrastructural features and haematology

**Figure 1:**
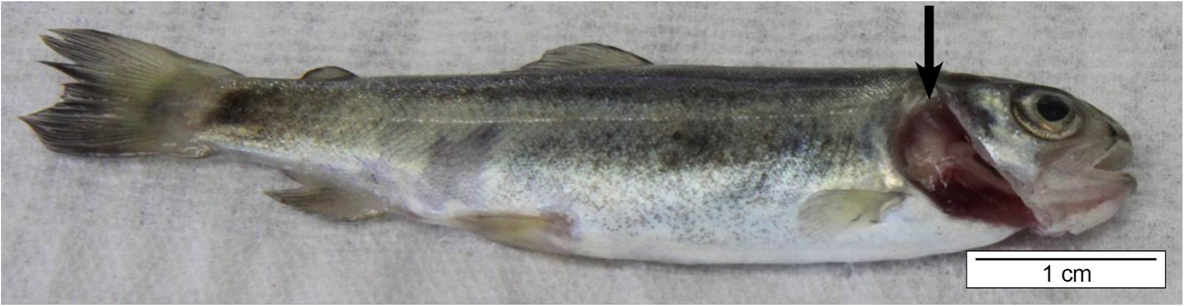
***F. columnare *elicited gill lesions in rainbow trout (*Oncorhynchus mykiss*) fry (operculum removed). **In young fish, the disease is mostly acute and the gill is the major site of damage. The lesions are exhibited by pale necrotic areas (arrow, bar = 1 cm).

**Figure 2:**
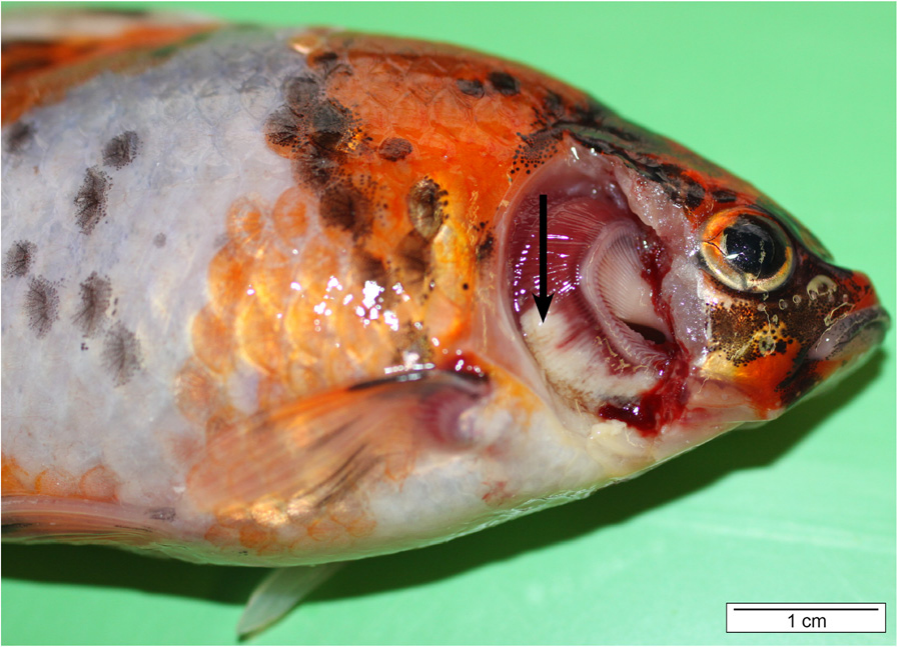
**Gill lesions in a shubunkin (*Carassius auratus*) (operculum removed) caused by *F. columnare. ***Yellowish-white areas of degeneration are visible in the ventral part of the first gill arch (arrow). When the *F. columnare *infection spreads rapidly throughout the gill lamellae, the fish may die in a short period of time without any other apparent lesions. Respiratory distress, caused by damage to the gills, appears to be the cause of death (bar = 1 cm).

*F. columnare* causes acute to chronic infections and typically affects the gills, the skin and fins. The clinical manifestation of columnaris disease amongst others is dependent on the virulence of the eliciting strain. In a study of Rucker et al., the strains of low virulence induced slow progressive infections at water temperatures above 21°C and caused massive tissue damage before death occurred [31]. Strains of high virulence caused fulminating infections and killed young salmon (*Salmo salar*) in 12 to 24 h at 20°C. Ordinarily, these fish did not show gross tissue damage at the time of death [31]. The same was found in a study of Pacha and Ordal [32] and Foscarini [33]. The gross pathology observed in the fish experimentally infected with strains of *F. columnare* of high virulence was usually very limited. Apparently, death occurred before gross external manifestations of the disease appeared. However, some of the last fish to die did show macroscopically visible signs [34]. Besides the virulence of the strain being a determinant factor, in coldwater and temperate fish, age also seems to have an important impact on the severity of the clinical signs. In young fish, the disease develops acutely and mostly damages the gills (Figure 1). In adults, the disease may adopt an acute, subacute or chronic course. When the disease course is acute or subacute in adult fish, yellowish areas of necrotic tissue can appear in the gills ultimately resulting in complete gill destruction (Figure 2) [1,32,35].

**Figure 3:**
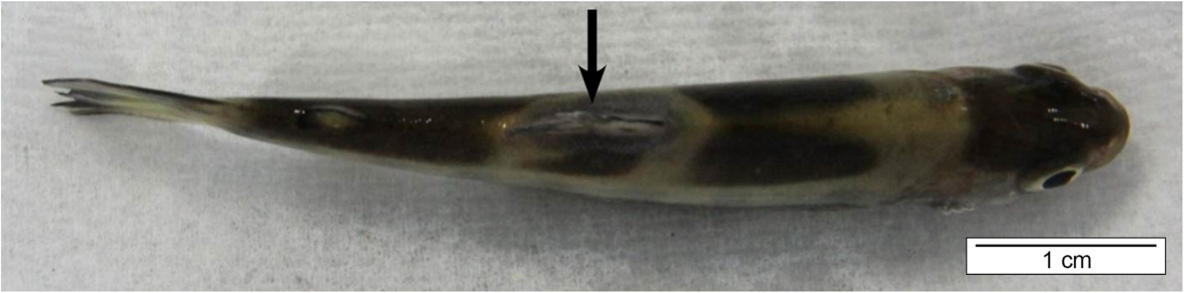
***F. columnare *induced a saddleback lesion (arrow) in rainbow trout fry. **The lesion is visible as a discoloration starting around its common location at the base of the dorsal fin and extending laterally to encircle the fish resembling a saddle. Hence, the descriptive term “saddle-back” is often used and the disease is denoted as “saddle-back disease” (bar = 1 cm).

In chronic cases, it takes longer before gill damage appears and skin lesions may develop as well [1,32,35]. On the body, small lesions start as areas of pale discolorations of the skin, which usually are surrounded by a zone with a distinct reddish tinge. This mostly begins at the base of the dorsal fin. Fin deterioration then occurs, starting from the lesion at the base of the fin and progressing to the outer edge, the opposite to normal finrot. The lesions then begin extending laterally from their common location at the base of the dorsal fin to encircle the fish resembling a “saddle-back” (Figure 3). The disease therefore is referred to as “saddle-back disease” [1,32,36]. Finrot is also often present [1,37].

**Figure 4:**
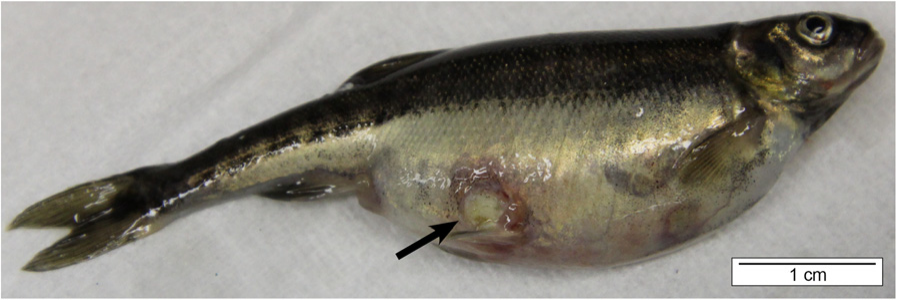
**Skin ulceration (arrow) in a minnow (*Phoxinus phoxinus*) caused by *F. columnare. ***The lesion has progressed into deeper skin layers, exposing the musculature. The edge of the ulceration displays a distinct reddish tinge and its centre is covered with yellowish-white mucus (bar = 1 cm).

**Figure 5:**
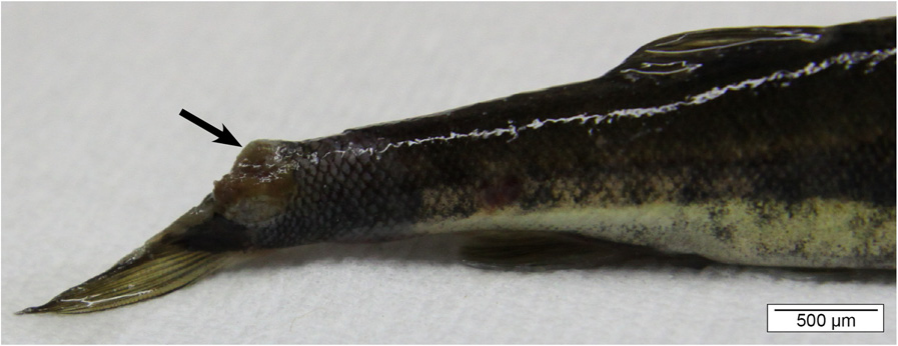
**Tail fin erosion in a minnow caused by *F. columnare. ***Columnaris disease led to complete disappearance of the upper half of the fin and exposing the underlying musculature (arrow, bar = 500 μm).

In rainbow trout, the area around the adipose fin may become dark and show erosions. These lesions expand to the peduncle, hence the name “peduncle disease” [1]. The lesions may progress cranially and caudally and even into the deeper skin layers, exposing the musculature leading to deep ulcers (Figures 4 and 5) [1,35]. The lesions typically are covered with yellowish-white mucus [1].

Ulceration of the oral mucosa also occurs, resulting in mouthrot. These mouth lesions are more lethal than are the skin lesions, since the painful oral lesions render the fish anorectic and lead to death due to starvation. Moreover, the disease spreads easily to the mandible and the maxilla. Secondary infections with fungi or other bacteria may deteriorate the situation and can be seen together with the filamentous bacteria [38]. In tropical fish, this clinical sign led to the disease being termed “cotton wool disease” or “mouth fungus” [1]. Finrot can also be present in tropical fish [37]. Lesions can be restricted to local skin discoloration, with or without ulceration, and degeneration of underlying muscle fibers [1,12,39].

The skin or gills need to be abraded, for bacteria to enter the bloodstream and cause systemic infections [40]. However, Hawke and Thune have isolated *F. columnare* from internal organs without any external lesions appearing [18]. Foscarini described that the pathological changes to the gill structure caused by columnaris disease go hand in hand with cardiac alterations [33]. The first day after infection, bradycardia was noticed with the formation of hyperplastic gill lesions. Degenerative processes of the lamellae in the following days resulted in a compensatory tachycardia. This research suggests that the interaction between the impaired gill vascular blood circulation and the cardiac changes could result in the death of the fish [33].

**Figure 6:**
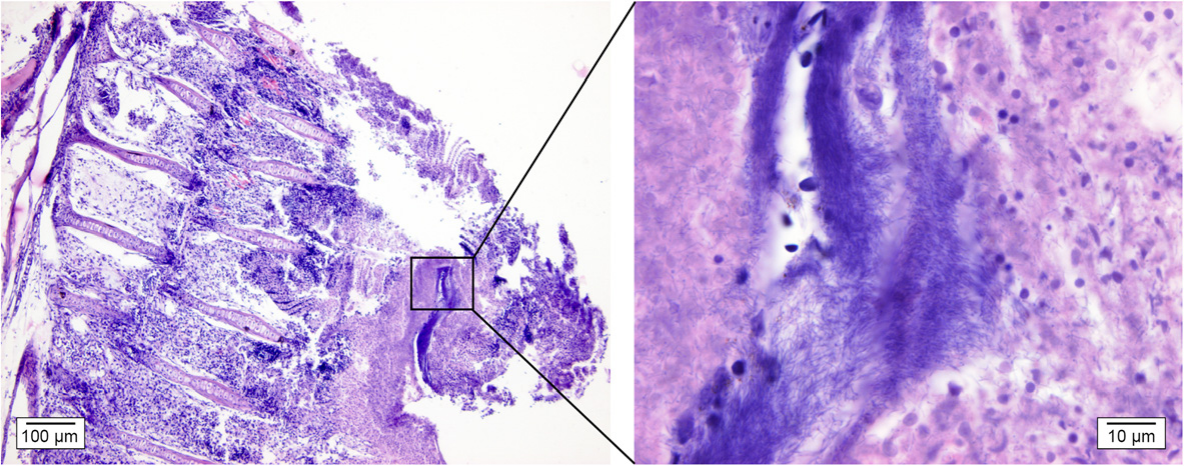
**Gill section of a koi carp infested with *F. columnare. ***In the left figure, extensive loss of branchial structures is visible. This is an advanced stage of the disease in which the filaments and the lamellae have fused and the gill epithelium is destroyed (H&E, bar = 100 μm). Complete clubbing of gill filaments may finally result in circulatory failure and extensive internal hemorrhage. A detail of this is depicted in the right figure, where *F. columnare *bacteria are visible as long, slender, purplish structures in between remnants of the gill tissue (H&E, bar = 10 μm).

Light microscopic examination of the affected gill tissue reveals the disappearance of the normal structure of primary and secondary filaments (Figure 6) [32,33,38,41]. In the initial phase, proliferation of epithelial cells of the gill filaments can be accompanied by an increase of mucous cells [33]. The proliferating tissue can occlude the space between adjacent gill lamellae. In more advanced stages, the occlusion can be total causing the gill lamellae to be completely surrounded by the propagating tissue. Congestion of gill lamellae occurs due to accumulation of blood masses and inflammatory cell infiltration can be noticed. Edema causes lifting of the surface epithelium of gill lamellae from the underlying capillary bed. In more advanced stages of the disease, fusion of gill lamellae and/or gill filaments appears [32,33,38,41]. Complete clubbing of gill filaments can finally result in circulatory failure and extensive internal hemorrhage [33]. Moreover, huge clusters of *F. columnare* can be found on the cell surface and/or in between necrotic sites (Figure 6, detail).

Columnaris disease may cause acute ulcerative dermatitis extending into the hypodermis and the muscle. Waterlogging can be present. The latter appears when the osmotic barrier is broken and thus forces water into the tissues, leading to severe dermal edema. Rupture of pigment cells with the loss of melanocytes can also be seen. Columns or hay-stack-like aggregates of bacteria can be gathered between dermal collagen fibers. The typical long and slender bacterial cells can easily be noted upon inspecting hematoxylin and eosin (H&E) or Giemsa stained sections from affected tissue where they appear bluish-purple and blue, respectively [38]. When all these changes occur rapidly, they may proceed to severe necrosis and sloughing of the epidermis [32,38].

**Figure 7:**
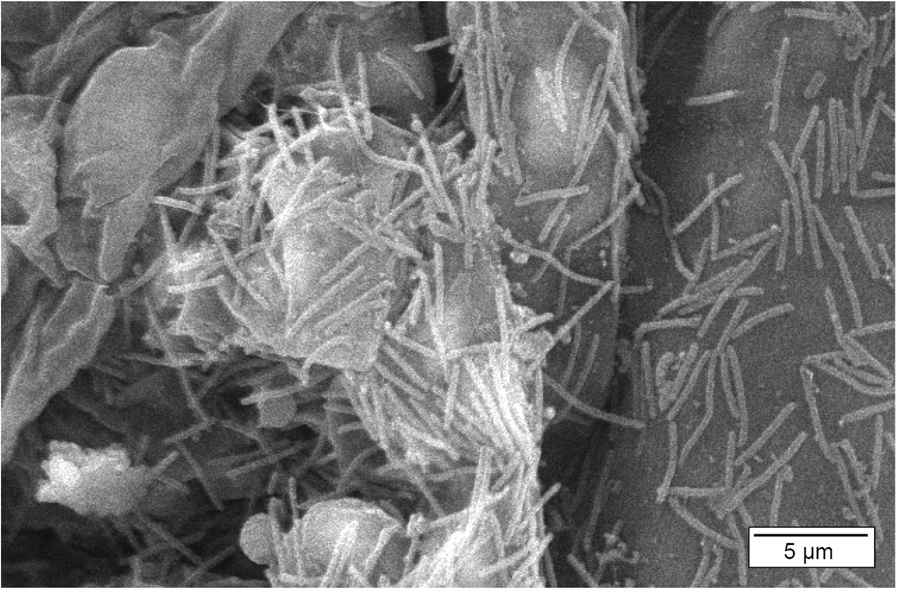
**Gill of a rainbow trout infested with *F. columnare. ***This scanning electron microscopic (SEM) picture of an affected gill arch reveals the presence of long, thin, rod-shaped bacterial cells, approximately 0.3-0.5 μm wide and 3-10 μm long (SEM, bar = 5 μm).

**Figure 8:**
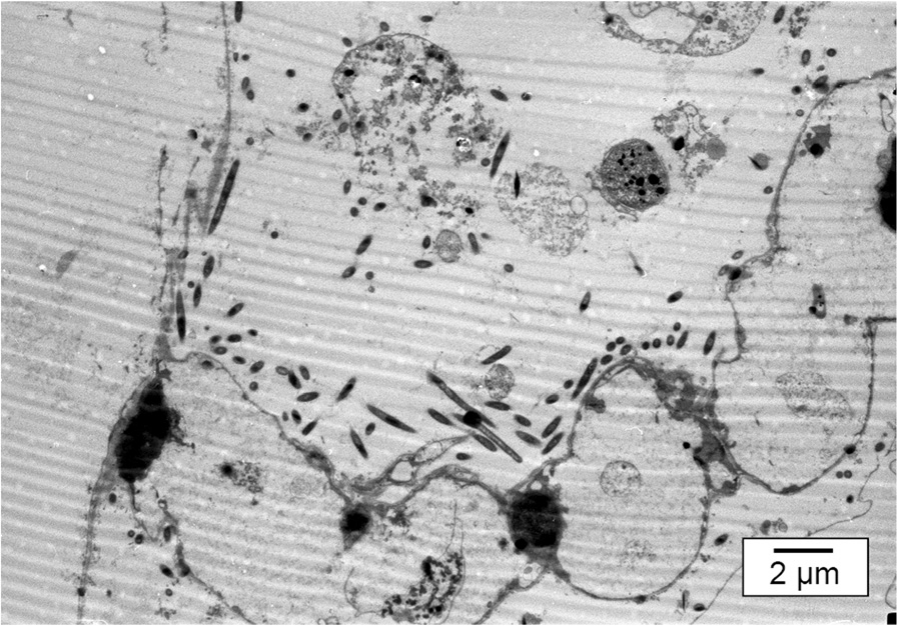
**Gill lamellae of a koi carp following infection by *F. columnare. ***This transmission electron microscopic picture clearly demonstrates the loss of structure of the gill lamellae. The *F. columnare *bacteria are lining up around the remnants of these lamellae, and are closely associated with the gill epithelium (TEM, bar = 2 μm).

Scanning electron microscopic (SEM) pictures of affected gill arches reveal the presence of rod-shaped bacterial cells, approximately 0.3-0.5 μm wide and 3-10 μm long (Figure 7). These long, thin bacteria adhere on the surface of the gills and appear to be aggregated rather than evenly distributed across the gill epithelium [42,43]. Transmission electron microscopic (TEM) examination of gill tissue shows numerous long, slender bacteria in close contact with the gill tissue (Figure 8).

Bullard and McElwain were the first to describe the ultrastructural features of saddleback lesions associated with experimental infections of *F. columnare* in channel catfish and zebrafish (*Danio rerio*) using SEM [44]. Channel catfish skin lesion samples had margins typified by shed epidermal cells and lesion centers that exhibited a multitude of rod-shaped bacterial cells, approximately 3-10 μm long and 0.3-0.5 μm wide, intermixed with cellular debris. Zebrafish skin lesion samples displayed a multitude of rod-shaped bacterial cells and exhibited similar ultrastructural changes. Scales were missing or, when present, denuded of epidermis.

In a cutaneous columnaris challenge model in koi carp, significant changes in blood parameters were observed in the infected fish [45]. For the hematologic parameters, a significant decrease was noted in Packed Cell Volume (PCV), haemoglobin concentration, red blood cell count, mean corpuscular volume and absolute lymphocyte counts. As for the biochemical parameters, marked hyponatremia, hypochloridemia and hyperglycemia were observed. Calcium and magnesium levels dropped only slightly and total serum protein and albumin-like protein concentrations decreased mildly. Alkaline phosphatase (ALP), aspartate aminotransferase (AST), lactate dehydrogenase (LDH) and creatine kinase (CK) secretions were significantly increased [45]. In a study by Řehulka and Minařík blood parameters from brook trout suffering from an acute, natural outbreak of *F. columnare* also revealed anemia, but levels of mean corpuscular volume and mean corpuscular haemoglobin were higher [15]. Total protein levels fell much below physiological parameters in infected fish. Calcium concentrations were reduced significantly. Blood Urea Nitrogen (BUN) measurements were much higher compared to normal levels. The catalytic activity of AST, alanine aminotransferases and LDH reached multiples of normal values. In contrast, ALP concentrations decreased. Hypoglycemia was noted. No data were shown on sodium or chlorine [15].

### 2.2 Diagnosis

Timely detection of this pathogenic agent is important to prevent its spreading and to reduce the economic loss to fish farmers.

The isolation of *F. columnare* is possible from external lesions, provided that the samples are taken from the edge of recent lesions [1]. *F. columnare* requires low nutrient media. No growth of *F. columnare* is reported on trypticase soy agar (TSA), nutrient agar or Marine 2216 agar [1]. In 1944, *F. columnare* was first cultured on Cytophaga agar, a nutrient poor medium [5]. Since then, several media, including Shieh [46] and TYES [47] have been developed in an attempt to improve growth of the bacterium. Growth does not occur in media that contain NaCl concentrations of over 0.5% or that have a pH lower than six [1]. Depending on the strain, the bacterium grows between 15 and 37°C [9,12], with an optimal growth occurring between 25°C and 30°C. Colonies appear after 24 to 48 h of incubation [12]. Glucose does not improve growth. Considering that *F. columnare* is easily overgrown by contaminating bacteria, selective media have been developed based on inherent resistance of *F. columnare* to different antimicrobial agents, including polymyxin and neomycin [6,8,25]. Decostere et al. added one μg/mL tobramycin to modified Shieh medium, demonstrating this to be an effective selective supplement for isolating *F. columnare*[41].

*F. columnare* largely displays two colony types on solid media; smooth and rhizoid [48]. Kunttu et al. characterized a third, rough colony morphology variant [49]. The rhizoid colony variants were assigned virulent and moderately adherent, the non-rhizoid rough colony variants non-virulent and highly adherent, and the smooth colony variants non-virulent and poorly adherent [49]. Colonies of *F. columnare* are notorious for their sometimes strong adherence to the agar. This trait is also exhibited in broth cultures where yellow, filamentous clumps of bacterial cells can form a thick ring at the surface of a glass recipient [12,50]. Adherence may be lost after several subcultures. Colonies can be recognized by their distinctive yellow pigmentation. The yellow color is due to the production of flexirubin pigments [1]. A method to identify the typical protein profile of *F. columnare* is whole-cell protein analysis [3]. Genomovar ascription has been performed using 16S-restriction fragment length polymorphism to divide *F. columnare* into three genomovars I-III [9]. Compared to RFLP, single strand conformation polymorphism (SSCP) would have an improved resolution power in the study of intraspecies diversity in *F. columnare*[51]. Polymerase chain reaction (PCR) has gained interest for definitive identification of *F. columnare* based on the selective amplification of the 16S ribosomal RNA gene using species-specific primers [52,53]. This technique gives a conclusive identification of the organism within a few hours and eliminates the need for biochemical testing which is laborious and sometimes inconclusive [52].

Besides isolation, other methods may be used for detecting this pathogen. These include serological methods such as the enzyme-linked immunosorbent assay [54] and the fluorescent antibody test [55,56]. Both have proven to be efficient and rapid for diagnosing columnaris disease. A loop-mediated isothermal amplification method (LAMP) for rapid detection of *Flavobacterium columnare* from infected fish organs (gills, skin and head kidneys) was established in channel catfish [57]. PCR may also be adopted, having the advantage of being able to detect very low levels of *F. columnare*[52,53]. In addition, Panangala et al. have developed a TaqMan-based real-time PCR targeting a 113 bp nucleotide region of the chondroitin AC lyase gene of *F. columnare*[58]. This PCR is specific, sensitive and reproducible for the detection and quantitation of *F*. *columnare* in tissues (blood, gills and kidney) of infected fish. Moreover, real-time PCR-based methods are distinctively more advantageous than conventional PCR as they eliminate the need for detection of amplified products by gel electrophoresis, thus reducing costs, time and labour [58]. Length heterogeneity PCR (LH-PCR) is based on comparing naturally varying lengths of 16S rDNA PCR products between bacterial groups [59]. LH-PCR has also proven effective in detecting *F. columnare* in fish tissue [60].

## 3. Pathogenesis

Various reports have noted the difference in virulence among *F. columnare* strains [11,12,40,42,61-65]. In the last decade, various studies have attempted to elucidate the pathogenesis of columnaris disease. Although significant progress has been made, still many questions remain unanswered on how this pathogenic organism elicits disease, and information concerning the bacteriological events preceding disease and death is scarce. Li et al. related this lack of knowledge to the absence of an efficient molecular manipulation system for *F. columnare*, especially a plasmid-based inframe knockout system [66], despite two recent reports on the establishment of genetic manipulation system for the bacterium [67,68]. Li et al. identified the type I restriction-modification system (R-M system) in *F. columnare* to improve electroporation efficiency and suggested that it would be of significant interest to examine the composition and diversity of R-M systems in strains of *F. columnare* in order to set up a suitable genetic manipulation system for the bacterium [66]. Only very recently, the full genome of *F. columnare* ATCC 49512 was sequenced [69].

### 3.1 Colonization

The colonization of the fish tissue is to be regarded as a complex multistep process which can be subdivided into the stages of attraction, adhesion and aggregation, requiring a step-by-step analysis. The exact factors mediating colonization have however not yet received the full attention they merit and to date remain largely unidentified.

The research group of Klesius et al. demonstrated by means of the traditional capillary tube method that the mucus from the skin and gills of catfish promotes chemotaxis of *F. columnare*[63]*.* The gliding motility of this bacterium is well known [1,12,41]. Indeed, the observation of a drop of bacteria grown in broth under a phase contrast microscope shows the slowly forward and backward gliding of *F. columnare*. Pate and Ordal described that the location of fibrillar structures spanning the gap between the outer membrane and the mucopeptide layer might play a role in the gliding motility of the *F. columnare* bacterial cells [70]. Although Klesius et al. acknowledged that they were not able to fully define the role that chemotaxis plays in the virulence of *F. columnare*, the chemotactic response of the more virulent genomovar ll isolates suggested that chemotaxis could be associated with virulence [63]. LaFrentz and Klesius developed a culture independent method to quantify the chemotactic response of *F. columnare* to skin mucus using blind-well chemotaxis chambers [71] generating similar results as stated above [63]. At least three carbohydrate-binding receptors (D-mannose, D-glucose and *N*-acetyl-D-glucosamine) associated with the capsule of *F. columnare* might be involved in chemotactic responses [62]. The gliding motility gene *gld*H was found to be significantly (*P* < 0.001) upregulated in *F. columnare* as soon as five minutes post-exposure to catfish mucus. When pretreated with D-mannose, there was no upregulation of gliding motility genes [62].

The ability to adhere is a prerequisite for the successful colonization of the host tissue. Decostere et al. performed a series of both in vivo and in vitro experiments and found that a highly virulent strain adhered more readily to the gill tissue of black mollies (*Poecilia reticulata*) than did the low virulence strain [11,12,42]. This research group underscored that the adhesion of *F. columnare* to the gill tissue constitutes an important step in the pathogenesis of columnaris disease. Bader et al. adopted an adhesion defective mutant of *F. columnare* in immersion challenge trials and found that the mortality was reduced with 75% and occurred 24 h later compared to the strains that still possessed the adhesion capacities, confirming the findings of the former research group [48]. In channel catfish genomovar II is considered to be more virulent than genomovar I [51,72]. Challenge of rainbow trout with genomovar I and II isolates of *F. columnare* demonstrated a difference in the cumulative percent mortalities (CPM), with the genomovar II isolates inducing significantly higher CPM [73]. Olivares-Fuster et al. compared adhesion of *F. columnare* genomovar I and II strains to the skin and gill of channel catfish and the gill of zebrafish (*Danio rerio*) [43]. At 0.5 h post-challenge, both strains adhered to the gill of channel catfish at comparable levels, but significant differences in adhesion were found in later datapoints over time. They concluded that particular strains of *F. columnare* exhibit different levels of adhesion to their fish hosts and that adhesion to fish tissues is not sufficient to cause columnaris disease. The same statement was previously made by Klesius et al. [63].

Kunttu et al. have shown that colony morphology affects the adhesion capacity of *F. columnare* to polystyrene and put forward the hypothesis that there is a link between virulence and rhizoid colony morphology [49,74]. The formation of different colony morphologies could be caused by changes in the cell surface components of the bacteria. It was also found that *F. columnare* changes colony morphology during experimental long-term storage in lake water, indicating the importance of different colony morphologies to bacterial survival [64,74,75]. Arias et al. did note that long-term starvation appears to decrease cell fitness and resulted in loss of virulence [75]. However, whether adhesion impairment was the cause of the observed virulence loss was not investigated.

*F. columnare* produces two types of mucus. The first one is an acidic polysaccharide and is made visible by ruthenium red staining. Another type of mucus is a basic, partially acetylated polygalactosamine, which cannot be stained with ruthenium red. Pate and Ordal described a capsular material which coated the surface of the bacterial cell that could be stained with ruthenium red [70]. They stated that the ruthenium red-positive material was probably an acid mucopolysaccharide that might be involved in the adhesive properties of the cells. The exact role of the mucopolysaccharides was however not further illuminated in this study. Decostere et al. demonstrated that the adherence capabilities of a highly virulent *F. columnare* strain to the gill were significantly reduced following treatment of the bacteria with sodium metaperiodate or incubating them with D-glucose, *N*-acetyl-D-glucosamine, D-galactose and D-sucrose [11]. Treatment with pronase or trypsin did not cause any significant inhibition of adhesion [11]. The same research group noted that the highly virulent strain had a thick capsule with a regular and dense appearance, whereas the capsule of the low virulent strain was much thinner. This made them to speculate that a lectin-like carbohydrate substance incorporated in the capsule might be partially responsible for the adhesion to the gill tissue.

Sun et al. conducted the first transcriptomic profiling of host responses to columnaris disease following an experimental immersion challenge by using illumina-based RNA-sequencing expression profiling [76]. A rhamnose-binding lectin (RBL) was detected as by far the most highly upregulated gene observed in their differentially expressed set, with expression increasing 105-fold by four hours following infection. This upregulation dramatically decreased at the later verified timepoints (24 h and 48 h), suggesting the importance of this gene during early infection events [76]. Immunohistochemical staining with antisera against an RBL in rainbow trout revealed the presence of these RBLs in mucous cells of the gill and in various cells related to innate immunity [77]. This expression pattern is consistent with the finding of *F. columnare* bacterial cell aggregates around goblet cells in common carp [11] and catfish [43] following experimental challenge with a highly virulent strain [76]. Beck et al. identified two distinct catfish families with differential susceptibilities to columnaris disease, with the one family completely resistant and the other susceptible [78]. In the susceptible family, an acute and robust upregulation in catfish RBL was observed following challenge that persisted for at least 24 h. After exposure of the catfish to different doses of the putative RBL ligands L-rhamnose and D-galactose, these sugars were found to protect the fish against columnaris disease, likely through competition with *F. columnare* binding of host RBL. Moreover, RBL expression was upregulated in fish fasted for 7 d (as compared to fish fed to satiation daily), yet expression levels returned to those of satiated fish within 4 h after re-feeding. These findings highlight putative roles for RBL in the context of columnaris disease and reveal new aspects linking RBL regulation to feed availability [79]. Further studies are needed to further pinpoint the (various) factor(s) responsible for mediating adhesion to the fish tissue.

The adhesion to the fish tissue was shown to be impacted by various environmental parameters. Using a gill perfusion model [61,80], Decostere et al. noted that the adhesion of a highly virulent strain to the gill tissue was enhanced by a number of factors, including immersion of the gill in divalent ion water, the presence of nitrite or organic matter, and high temperatures [61]. The positive effect of high temperatures on the adhesion has also been demonstrated by Kunttu et al. [49]. Adhesion seems to decrease in vitro as salinity goes up [79]. Expression of adhesins by bacteria is regulated on two different levels. One is directed by environmental sensing and transcription of specific regulatory elements, and the other is a random switching on and off of adhesion genes by submission of the bacterial population to unpredictable environmental conditions [81]. If and how both phenomena are established in *F. columnare* remains to be elucidated.

An aggregative adhesion pattern of a highly virulent *F. columnare* strain onto gill tissue is a distinct feature in both in vivo and organ culture experiments [35,42,53,61]. This results in an irregular gill surface covered by a thick mat consisting of numerous clumps of *F. columnare* bacterial cells, most likely impeding oxygen uptake and causing death of the fish. These microcolonies are not observed when a low virulence strain is used. Biofilm formation capacity was demonstrated in vitro for one *F. columnare* strain when exposed to mucus [67]. These features point towards biofilm formation potentially being an important stage in the pathogenicity of *F. columnare*, warranting its further investigation.

The developmental switch to the biofilm state is commonly regulated by quorum sensing. Quorum sensing (QS) is a mechanism of gene regulation in which bacteria coordinate the expression of certain genes in response to the presence or absence of small signal molecules. In many Gram-negative bacteria, the signal molecule is an *N*-acylhomoserine lactone (AHL) [82]. Additionally, a signaling molecule known as autoinducer-2 (AI-2) may also be employed [83]. Wagner-Döbler et al. found short-chain AHL-type activity in *Flavobacterium* sp., but no AHL-presence could be confirmed using gas chromatography-mass spectrometry (GS-MS) [84]. Using light chromatography-mass spectrometry (LC-MS), Romero et al. described the presence of short-type AHL activity in the culture media of nine *Tenacibaculum maritimum* strains, biofilm-forming members of the phylum *Bacteroidetes*, formerly referred to as “*Cytophaga-Flavobacterium-Bacteroides*” group [82]. To our knowledge, so far, QS by AHL or AI-2 has not yet been demonstrated in *F. columnare*.

### 3.2 Exotoxins, bacteriocins and endotoxins

It is known that polysaccharide degradation in combination with the secretion of various extracellular enzymes participate in the destruction of skin, muscle and gill tissue [1], enhancing pathogenicity. In culture, *F. columnare* produces an enzyme that degrades chondroitin sulfates A and C and hyaluronic acid, the complex polysaccharides of connective tissue. This so-called chondroitin AC lyase acts specifically on a group of acidic mucopolysaccharides found primarily in animal connective tissue [40]. No correlations were found between host origin, geographic distribution, and amount of enzyme produced by different isolates [85]. AC lyase is alleged to play a role in the virulence of *F. columnare*[49,65]. Though high AC lyase activity solely would not be enough to induce virulence in *F. columnare* strains, both high AC lyase activity and gliding motility of the bacteria would be needed for *F. columnare* to be virulent [49]. Proteases also contribute to damaging the tissue or enhancing invasive processes [40]. Newton et al. isolated and partially characterized proteases of 23 isolates of *Flavobacterium columnare* derived primarily from channel catfish raised in the southeastern United States [86]. The bacterial isolates were divided into two groups according to the apparent molecular masses of proteases after zymographic resolution by non-reducing, non-denaturing sodium dodecyl sulfate–polyacrylamide gel electrophoresis (SDS–PAGE) with gelatin as the protease substrate. Isolates of group one, produced two proteases with apparent molecular masses of 53.5 and 58 kilodaltons. The isolates of group two, revealed three proteases with apparent molecular masses of 44.5, 48 and 59.5 kilodaltons. All isolates degraded gelatin and casein. Seven out of 23 isolates degraded elastin. More protease was produced in a medium with low nutrients and salts than in media with higher concentrations of nutrients. Moreover, a sharp increase was seen in protease production during the first 24 h of incubation and these levels dropped only slightly in the remaining days of the experiment [86].

Bacteria not only need to enter the host tissue, they also need to eliminate competitive bacteria. Different strains of *F. columnare* release specific, non-transmissible, bactericidal substances equivalent to colicins of *Escherichia coli* into the environment to reduce competition from other bacterial strains [87]. It was postulated that cells of *F. columnare* also possess multiple specific receptors for the bacteriocins, and, consequently, the cells are sensitive only to those bacteriocins for which the cell possesses receptors [87]. Antagonism of *Pseudomonas sp.* MT5 against *F. columnare* bacteria was found to be very strong in agar assays [88]. However, antagonistic baths of the *Pseudomonas* bacterial strain could not yet prevent nor treat a *F. columnare* infection following experimental challenge [23].

Differences in LPS composition between highly and low virulent strains of *F. columnare* retrieved from channel catfish have been detected [89]. Analysis of LPS by immunoblotting revealed that an avirulent mutant of a *F. columnare* isolate lacked the high molecular weight components of LPS present in virulent isolates. Based on these differences of LPS and total protein profiles, the research group was able to discriminate the attenuated mutant from other *F. columnare* strains [89]. Kunttu et al. determined LPS-profiles of different colony morphology variants of *F. columnare* in rainbow trout [49]. Colony morphology variants of the same strain produced a similar single LPS band. However, there were size differences between different strains. Both research groups used different LPS extraction and detection methods, rendering comparison of the obtained results impossible [49].

### 3.3 Interaction with the fish immune system

When encountering an infection, the first system activated is the innate immune system. It is assumed that the surface mucus layer, as a first physical-immunological barrier, plays an important adhesive role and that it is part of the innate host resistance of fish to disease [43]. Antibacterial characteristics of the fish mucus against *F. columnare* have been demonstrated. In an experimental challenge trial of cutaneous columnaris disease in koi carp [45], lesions were noted on locations where mucus had been removed. After incubating *F. columnare* inoculated agar plates, a lower number of colonies was counted on plates to which mucus was added. Fluorescent microscopy of a stain-based bacterial viability assay also revealed a higher number of dead bacteria in *F. columnare* cultured with mucus [45]. Staroscik and Nelson compared growth, biofilm formation, extracellular protease production and changes in protein expression of a highly virulent *F. columnare* strain cultured in media supplemented with juvenile Atlantic salmon (*Salmo salar* L.) skin mucus with the same media without mucus [67]. Interestingly, and in contrast to the reasoning resulting from the above mentioned studies, salmon surface mucus promoted the growth of *F. columnare*, induced the bacteria to grow as a biofilm and increased extracellular protease activity [67]. This might indicate that skin mucus of different fish species responds differently to the bacteria, or that it is the *F. columnare* strain which is critical in determining the antibacterial capacities of the mucus.

The fish skin in itself also forms a barrier against pathogens. Aranishi et al. immersed Japanese eel (*Anguilla japonica*) in a *F. columnare* suspension to monitor dermal nonspecific stress responses [90]. They found that cathepsins B and L activities in the infected fishes increased more than 1.5-fold over their initial values over a 48 h period, along with a 4.5-fold increase in bacteriolytic activity. These cathepsins likely participate in bacteriolysis associated with Japanese eel skin and their activities may represent an important nonspecific response of eels [90].

Some studies reveal that *F. columnare* might be able to avoid parts of the immune system. In a study by Ourth and Bachinski, the catfish alternative complement pathway (ACP) was inhibited by large amounts of sialic acid contained by Gram-negative bacterial pathogens, including *F. columnare*[10]. Sialic acid seemed to be the determining factor for the pathogenicity of *F. columnare*, as very little or no bactericidal activity was produced against this bacterium by the catfish ACP. Furthermore, the latter greatly increased after removal of sialic acid with neuraminidase [10]. Another recurrent feature in *F. columnare* infections, is the lack of an inflammatory response as observed upon inspecting affected tissues microscopically. This resulted in the hypothesis that *F. columnare* triggers the endogenous programmed cell death machinery of immune cells to evade the immune system. Do Vale et al. have already proven that apoptosis can be a very powerful pathogenic strategy by inducing lysis of phagocytic cells [91]. Sun et al. speculated that negative regulation of one of the central innate immune signaling pathways NF-κB, may be the result of immune evasion or manipulation by *F. columnare* via secreted toxins [76]. Furthermore, high levels of inducible nitric oxide synthases (iNOS), apoptotic-promoting interferon (IFN) and other members of oxidative stress responses and apoptotic pathways, such as caspase 8 and G3BP1 (Rasputin), were observed following a *F. columnare* infection. Rasputin plays an inhibitory role in negative regulation of apoptosis and was highly downregulated at all examined time-points [76]. This could explain why hardly any inflammatory cells appear in early infections with *F. columnare* as was described by Morrison et al. [36].

Ourth and Wilson demonstrated that *F. columnare* is resistant to the bactericidal action of serum of non-immunized catfish via the ACP [92]. Ourth and Bachinski confirmed this finding [93], but also demonstrated that the classical, antibody-mediated complement pathway is highly effective in killing *F. columnare*[10]. The virulence of *F. columnare* can also be influenced by transferrin [94]. The survival time of fish experimentally challenged with *F. columnare* by intraperitoneal injection with iron-free human transferrin (Sigma) was reduced when iron was injected prior to exposure. The effect of iron was only evident in one of the two strains examined when the challenge was delivered via immersion. These results indicate that iron depletion may limit the virulence of *F. columnare* more in systemic infections than in external infections. This hypothesis is supported by data indicating that administration of transferrin prior to challenge increased survival after challenge by injection but had little or no effect on bath-challenged fish [94]. However, the results found were not consistent for all strains tested and no statistical data were presented.

Several immunization experiments adopting different administration routes, have proven that fish can be protected from subsequent *F. columnare* infections by activating the adaptive immune system [20,22,95-98]. High agglutinin titers and good protection were obtained in trout following subcutaneous or intraperitoneal injection with heat-killed *F. columnare* cells [22]. Schachte and Mora obtained a high agglutinin titer in channel catfish by intramuscular injection of heat-inactivated cells of the pathogen, but the actual level of protection was not examined [99]. Becker and Fujihara reported that rainbow trout injected with heat-killed cells produced an agglutinating titer of 1:5120, and that 60-70% of the trout later survived an injection of 10^6^ live *F. columnare* cells [96]. Tilapia (*Oreochromis niloticus* (L.)) could mount a significant humoral response in plasma and cutaneous mucus to *F. columnare* after intraperitoneal immunization with formalin-killed sonicated cells in Freund’s complete adjuvant [97]. Protection levels were not investigated in the latter study. Protection was obtained after oral immunization with heat-killed or formalin-killed cells of *F. columnare* in the fish feed of three-month-old coho salmon (*Oncorhynchus kisutch*) [22,100]. However, the protection as observed in these studies did not go hand-in-hand with high agglutinin titers. Bath-immunization with a bacterin was shown to protect carp against experimental challenge but antibodies against *F. columnare* were not detected in sera from immunized fish [101]. Song’s work with bacterins demonstrated unequivocally that immersion vaccination could result in high levels of protection, but field test results were inconsistent [102]. Song was able to demonstrate that there was cross-protection between isolates and that there may be a common protective antigen among the strains tested [102]. Polyvalent vaccines have also been tested. Using intraperitoneal injections of a combination of formalin-killed *F. columnare*, *Aeromonas salmonicida* and *Yersinia ruckeri* antigens, interference from *A. salmonicida* antigen was shown to suppress responses to two other antigens [100]. Commercially available oral and bathing vaccines have been successfully tested in largemouth bass (*Micropterus salmoides*) fry and salmon, respectively [95,103]. Vaccination trials are further elaborated on below.

## 4. Importance of environmental factors

Karvonen et al. described the effect of global warming on the prevalence of different fish parasites and bacteria [104]. *F. columnare* could be one of the many taking advantage of this phenomenon. Indeed, transmission of columnaris disease is more efficient in higher temperatures [30,105]. Holt et al. found that when steelhead trout (*Salmo gairdneri*) or coho salmon (*0. kisutch*) experimentally infected with *F. columnare* were held in water at 12 to 20°C, mortality increased with temperature [106]. As stated above, adhesion to gill tissue of a highly virulent *F. columnare* strain is enhanced at increased temperature [61] and chondroitin AC lyase activity of this pathogen increases along with the temperature [107]. The influence of rearing density and water temperature in rainbow trout was studied by Suomalaien et al. [105]. Normal rearing densities with high temperatures (23°C) proved to increase both transmission rate of columnaris disease and mortality in the fish. Normal densities at low temperatures (18°C) did not affect mortality, but increased the transmission rate of columnaris disease [105].

Columnaris disease is furthermore influenced by water quality. Decostere et al. observed significantly higher bacterial titers on the gills when organic matter or nitrite were added to an organ bath when performing ex vivo trials with *F. columnare*[61]. They discussed that organic matter could concentrate nutrients to feed the bacteria and that degrading enzymes could be kept in close contact with the host tissue. Bacterial titers were furthermore markedly lower in gills placed in an organ bath with distilled water with or without 0.03% NaCl compared to the titers of gills suspended in Ringer solution or in formulated water containing divalent ions (magnesium and calcium) [61]. Morris et al. noted that the survival of the fish exposed to *F. columnare* significantly increased as unionized ammonia concentrations increased [108]. These results suggest that complex interactions can complicate prediction of the responses of fish to concurrent chemical stressors and bacterial pathogens [108]. Bandilla et al. described that co-infections of ectoparasites with *F. columnare* increased the susceptibility of rainbow trout to the bacterial pathogen [109]. Compared with single infections, the mortality was significantly higher and the onset of disease condition occurred earlier in fish which were concomitantly infected by the parasite *Argulus coregoni* and *F. columnare*[109].

## 5. Experimental trials/pathogenicity tests

A reliable and reproducible experimental infection model is crucial for studying bacterium-host interactions and evaluating the efficacy of both curative and preventive measures. Most researchers recognize, in experimental challenges with *F. columnare,* the fine and delicate balance and complex interplay between the bacterial cells, fish and environment in the successful reproduction of columnaris disease.

Hitherto, columnaris disease has been reproduced experimentally in black mollies (*Poecilia sphenops*) [12], channel catfish [43,44,110,111], eel [94], golden shiner (*Notemigonus crysoleucas*) [112], koi carp [45], rainbow trout [49,65,105,113], tilapia [94] and zebrafish [43,44].

In the various experimental infection trials, several ways to both cultivate and harvest the bacterial cells for inoculation were adopted. Different media were used to cultivate the bacteria, including Hsu-Shotts or modified Hsu-Shotts medium [79,114], Ordal’s medium [115], Anacker and Ordal’s medium also referred to as “Cytophaga medium” [94], *F. columnare* growth medium broth [115] and Shieh or modified Shieh medium [12,23,43-45,105]. The bacteria were mostly grown on a shaker [43-45,75,109-111]. Temperatures and incubation times of the bacteria varied from 21°C to up to 30°C and from 24 to 48 h, respectively.

To reproduce columnaris disease experimentally, largely two inoculation routes were adopted, viz. bath (immersion) and injection. With regard to immersion challenges, mostly full-grown broth cultures were added to the water with water temperatures of the immersion water varying from 18°C to 30°C [12,23,42-44,111]. Only in a minority of cases were the bacterial cells first harvested through centrifugation and consequently resuspended before being added to the inoculation bath [33,45,114]. Times during which the fish were bath exposed varied from 15 min [13,72,105] over 30 min [12,23,43-45,109] to 45 min [42], one hour [79,94] and longer [110,111]. When the bacterial cells were administered through injection, then they were retrieved from centrifuged broth cultures following discard of the supernatant [12] or immediately taken from a full-grown broth culture without primary centrifugation [13,58,94]. A third inoculation route consisted of adding dead fish that were first injected intraperitoneally with a virulent *F. columnare* strain, to the aquarium water in which fish were immersed [20]. In some researches, the successful reproduction of columnaris disease depended on or the severity of the elicited disease increased by abrading the skin or gills of the fish [45,94,105] whereas in another study, clipping the fin had no effect on success after immersion challenge with *F. columnare*[12].

The infection route, which determines the way the bacterial cells are to be grown/collected, also defines the disease producing capacity of the adopted strains and the disease picture they elicit. Indeed, especially for low virulent strains, higher morbidity and mortality rates were noted after injection of fish as compared to inoculation through immersion [12]. Pacha and Ordal reported a similar finding [34]. They stated that contact with highly virulent strains induced infection and disease contraction more than intramuscular injection, whereas injection of low virulent bacteria more readily induced infection and disease contraction than did contact [34].

Environmental conditions can also highly influence morbidity and mortality rates during a challenge trial. To illustrate the dramatic effects of water temperature on the level of mortalities, the investigation of Holt et al. is particularly relevant [106]. This team challenged steelhead trout (*Oncorhynchus mykiss*), Chinook salmon (*O. tshawytscha*) and coho salmon with *F. columnare*, via the water-borne route. At a water temperature of 9.4°C, there were no mortalities attributable to *F. columnare*. By increasing the temperature to 12.2°C, mortality became as high as 4-20%; at 20.5°C, all the steelhead trout and coho salmon and 70% of the Chinook salmon died [106]. High (23°C) rearing temperatures also increased mortality significantly in rainbow trout compared to lower (18°C) temperatures [105]. Fish density at fish farms is also a key player in influencing mortality in an outbreak of columnaris disease as mortality rates started earlier and remained higher when fish were stocked at high densities [105]. Water flow is another important factor since it acts as a determining factor with regard to the contact time between the possibly present bacteria and the host tissue. High mortality rates were observed in elvers kept in standing water, while in aquaria with running water, mortality in elvers was reduced by half [94].

## 6. Control

### 6.1 Preventive measures

Management plays a key role in the prevention of the disease. Cunningham et al. showed that some commonly recorded production variables (feed consumption, pond depth, ammonia levels and stocking events) were associated with columnaris disease outbreaks and, if monitored, could help identify “at risk” ponds prior to disease outbreaks [116]. Suomalainen et al. pointed out that reduction of fish density could be used in the prevention of columnaris disease especially if water temperature is high [105]. As lower rearing density can also decrease the transmission of ectoparasites and penetrating endoparasites, it could be an efficient tool in ecological disease management as a whole [105]. High nitrite levels and organic load can stimulate the adherence capacity of *F. columnare*[61], and therefore it is important to control these parameters as well. Furthermore, water treatment could aid in averting a bacterial outbreak. Conrad et al. reported that ozone treatment of water significantly reduced the numbers of added *F. columnare*, which could be a practical method of prevention [117]. Salt and acidic bath treatments could be used to disinfect water contaminated by *F. columnare*[23]. An in vivo immersion challenge of *F. columnare* in channel catfish and goldfish (*Carassius auratus* L.) revealed decreasing mortality as salinity goes up, with significantly lower and no mortalities when salinity reaches values of 1.0‰ and between 3 and 9‰, respectively [79]. If the fish can be adapted to salt levels of at least 1.0‰, this method could be used as a possible preventive measure in columnaris disease. Shoemaker et al. suggested that in the absence of natural food, juvenile channel catfish should be fed at least once every other day to apparent satiation in order to maintain normal physiological function and improve resistance to *F. columnare*, since deprivation reduced innate resistance of catfish to columnaris disease [118].

Besides optimizing and adjusting management practices, chemical agents can also be adopted as a preventive approach. Davis concluded that the development or intensification of columnaris disease could be prevented by treating the fish for 20 min in a copper sulfate (CuSO_4_) bath at 37 mg/L (1:30 000) or by adding copper sulfate to pond water at 0.5 mg/L [4]. Dipping the fish one at a time in a 1:2000 copper-sulfate for one to two minutes was also proven to be effective in the prevention of the disease. Rogers suggested the addition of potassium permanganate (KMnO_4_) to the water at 2 mg/L [119]. Darwish et al. also confirmed the prophylactic value of KMnO_4_ at doses around 2 mg/L [120]. Prophylactic treatment of channel catfish with 15 mg/L chloramine-T reduced fish mortality from a *F. columnare* infection from 84–100% to 6–14% [121]. Thomas-Jinu and Goodwin demonstrated the efficacy of prophylactically given oxytetracycline against mortality in channel catfish and also reported zero mortality for the combination of sulphadimethoxine and ormetoprim in feed prior to bacterial challenge with four highly virulent strains of *F. columnare*[111].

Another method to prevent columnaris disease is through vaccination. Although vaccination trials have not always been successful, success rates have increased as knowledge on fish immunity and its role in the defense against bacterial diseases continues to expand. Bath immunization with a bacterin was shown to protect carp against experimental challenge, but no agglutinin could be found in sera from immunized fish [101]. Immersion of channel catfish in a bacterin, when performed each year, induced a significant decrease in mortality compared to unvaccinated fish [98]. Fujihara and Nakatani obtained protection against columnaris disease in 3-month-old coho salmon by oral immunization with heat-killed cells of *F. columnare* incorporated into fish feed [22]. Ransom proved that prolonged feeding (over three months) of formalin-killed bacteria provided high levels of protection [100]. Ourth and Bachinski proposed that strains containing sialic acid could serve as potential vaccine strains for columnaris disease [10]. As stated above, immunization with formalin-killed sonicated cells in Freund’s complete adjuvant injected intraperitoneally in tilapia resulted in a significant systemic humoral response within two weeks and antibody levels almost tripled following secondary immunization [97]. At 10 weeks postimmunization, the mean antibody titer remained significantly elevated. Antibodies were also observed in cutaneous mucus of these fish at six and eight weeks postimmunization [97]. An attenuated immersion vaccine currently is registered for the use in channel catfish in the USA [20]. Fry between 10 to 48 days post hatch that were vaccinated through immersion achieved a relative percent survival (RPS) between 57 and 94% following *F. columnare* challenge. This vaccine was also proven to be efficient in largemouth bass fry resulting in RPS values between 74 and 94%, depending on the vaccine dose [20]. Bebak et al. tested a commercial oral vaccine in largemouth bass fry [95]. Vaccinated fish had a 43% lower risk of death by *F. columnare* during the field trial [95]. An immersion vaccine consisting of a bacterin of *F. columnare* was also brought to the market in the USA as an aid in the prevention of columnaris disease in healthy salmonids of over three grams [103].

Probiotics appear to be a promising way in the prevention of different bacterial diseases in aquaculture [122]. Boutin et al. isolated different strains of commensal bacteria from the skin mucus of unstressed brook charr (*Salvelinus fontinalis*) which in vitro revealed antagonistic effects against *F. columnare*[123]. The strains were mixed and used to treat columnaris disease in an in vivo experiment. This resulted in a significant decrease of mortality indicating the potential use of these probiotic candidates in the efficient and durable management of columnaris disease. The immunostimulants β-glucan and β-hydroxy-β-methylbutyrate raised the levels of immune function parameters, but did not improve survival in challenge trials with *F. columnare* at any concentration of the stimulants used [113]. The research group of Sink et al. demonstrated that mortality rates in golden shiners fed high-fat diets with a dairy-yeast prebiotic were significantly lower after a challenge trial with *F. columnare*[112].

Recent studies have demonstrated genetic variation in resistance towards *F. columnare*[73,78,124]. Arias et al. presented experimental data on the susceptibility to columnaris disease of hybrid catfish (female channel catfish × male blue catfish (*I. furcatus*)) (C×B) [124]. Under experimental conditions, C×B hybrids were significantly more resistant to columnaris disease caused by a highly virulent strain of *F. columnare* belonging to genomovar II compared to channel catfish and blue catfish. Beck et al. also found one of the two investigated catfish families to be completely resistant towards *F. columnare* resulting in no mortality after inoculation with the bacteria [78]. These *F. columnare* resistant families could be of great financial importance in the catfish industry. Interestingly, LaFrentz et al. found that the two families that exhibited the highest CPM after *F. columnare* challenges, had the lowest CPM following *E. ictaluri* challenge, the latter being the most prevalent bacterium to cause disease and mortality in the catfish industry [73]. Further research on larger numbers of families is needed to determine whether there is any genetic correlation between resistance to *E. ictaluri* and resistance to *F. columnare*, the two leading bacterial diseases in the catfish industry [125].

### 6.2 Curative approach

Treatment of columnaris disease using antimicrobial agents has known different success rates. External treatments are possible only in early stages of the disease, when the infection is still superficial [6]. Drugs which have been used effectively in bath therapies are chloramphenicol [126], nifurpirinol [127,128], nifurprazine [129,130] and oxolinic acid [131,132]. If the disease is in an advanced stage and/or signs of septicaemia are observed, it is necessary to administer antimicrobials in the feed. Oxytetracycline given orally for up to 10 days proved effective in early as well as advanced outbreaks of columnaris disease in Pacific salmon (*Salmo salar*) [6,133]. Lack of success of orally administered oxytetracycline has also been reported [134]. Sulfonamides, such as sulfamerazine and sulfamethazine, can be used orally but would be less effective than other drugs [6,135]. Nitrofuran can also be administered orally for 3 to 5 days [6,96,130]. Gaunt et al. demonstrated the efficacy of florfenicol in the feed against columnaris disease in channel catfish [136]. Darwish et al. also illustrated the clear benefit of florfenicol against a mixed infection of *A. hydrophila* and *F. columnare* in Sunshine bass (hybrid striped bass, *Morone chrysops* female × *Morone saxatilis* male) [115]. The excessive use of antimicrobial agents to withstand *F. columnare* has its negative attributes though [20]. These include possible allergic reactions elicited in the user after food contact [137]. Potential impacts on human health resulting from the emergence of drug-resistant bacteria and the associated risk of transfer of these resistant traits to the environment and human-associated bacteria are also a major concern [137]. Declercq et al. demonstrated the in vitro multiple resistance of *F. columnare* strains originating from ornamental fish toward several clinically important antibiotics, such as quinolones and tetracyclines [138]. The results obtained in this study appeal for less prudent use of antimicrobials especially in the ornamental fish industry and therefore urges to limit their use and to focus on the development of alternative curative and preventive measures against columnaris disease.

Besides resorting to antimicrobial agents, chemicals have also been adopted in the curative treatment of columnaris disease. In a study by Thomas-Jinu and Goodwin, the herbicide Diquat^®^ (Zeneca Agricultural Products, Wilmington, DE, USA) was shown to significantly reduce channel catfish mortalities to zero percent after challenge with *F. columnare*[111]. The herbicide has also proven to be effective in the treatment of columnaris disease in salmonids [6,139]. Copper sulfate [4] and potassium permanganate [119] are among the older chemicals used for treatment and prevention of columnaris disease in pond fishes. The organic load in water affects the efficacy of potassium permanganate, but methods are available to estimate that organic load and compensate by adjusting the level of the chemical [6]. Darwish et al. suggested that copper sulfate has clear therapeutic value against *F. columnare* infections in channel catfish when treated in an ultralow flow-through system during 4 h [110]. Thomas-Jinu and Goodwin on the other hand proved the inefficacy of this same chemical against columnaris disease, which might be due to the advanced stage of the experimental infection at the time of treatment [111]. An in vitro assay of commercial products containing peracetic acid was proven to be effective against *F. columnare* infection [140]. Laanto et al. reported some *Flavobacterium* sp. phage lysates to inhibit growth or lyse the bacterial cultures [141]. The authors recommended that the causative agent of this strong inhibition or lysis should be studied further for the possibility of developing antimicrobial agents. Prasad et al. also described the successful use of *F. columnare* phage FCP1 to combat columnaris disease in walking catfish (*Claries batrachus*) [142]. Phage treatment led to disappearance of gross symptoms, resulted in a negative bacteriological test, a detectable phage, and 100% survival in experimentally infected *C. batrachus*. The result of this study opens new perspectives for the treatment of columnaris disease elicited by antimicrobial resistant *F. columnare* strains [142].

## 7. Conclusion

Despite the worldwide importance of columnaris disease and the multitude of studies focusing on its causative agent, only snippets of the bacterium-host interactions have hitherto been exposed. This results in major knowledge gaps still prevailing on amongst others how the pathogen is able to establish and maintain a grip on the skin and gill tissue and elicit disease and mortality. Methods for rapid identification of the bacterium in the environment, host tissue or in culture have indeed evolved rapidly and some techniques permit to trace even a few bacterial cells. However, the rationale for the difference in virulence among *F. columnare* strains remains to be unraveled. Full genome sequencing of the reference strain has been an important step towards gaining insight into the pathogenesis of columnaris disease. Efficient molecular manipulation systems could be of great importance to further explore this domain. Genetic knockouts could in effect illuminate virulence factors involved in the pathogenesis of this disease. Although various models to experimentally induce columnaris disease are available, there is a lack of a standardized experimental model hampering the comparison of retrieved data in between research groups and the drawing of consistent conclusions. Consistency in the cultivation protocol of the bacteria in terms of incubation temperature, infection dose, exposure time and infection route is desirable. Extrapolation of a standardized model from one to another fish species is not possible rendering it necessary to set-up an experimental model for each fish sensitive species. Besides further elucidating the pathogenesis of columnaris disease, the development of efficient curative and effective preventive measures also needs to continue. With the upcoming antimicrobial resistance, focus should be laid on the search for preventive measures. Management plans trying to optimize or adjust fish densities and control water quality parameters are a first critical step in controlling columnaris disease. Vaccines, chemotherapeutics and probiotics could possibly have a promising future in the prevention and control of the disease as some studies have already demonstrated. Another interesting envisaged method to mitigate columnaris disease is the creation of a fish breeding program introducing *F. columnare* resistant fish. Data generated through pathogenesis studies will allow for the further development and optimization of cost-effective and environmentally friendly prevention methods of columnaris disease in the various susceptible fish species.

## 8. Competing interests

The authors declare that they have no competing interests.

## 9. Authors’ contributions

AM Declercq: Gathering information and papers, writing the outlines, writing the paper; A Decostere: Proposing the subject, reviewing and editing the paper; F Haesebrouck: Critically reviewing the manuscript; W Van den Broeck: Interpretation of histological, SEM- and TEM- pictures, critically reviewing the manuscript, P Bossier: Critically reviewing the manuscript. All authors agreed on outlines and the final version of the paper.
